# Proteomic Characterization of a 3D HER2+ Breast Cancer Model Reveals the Role of Mitochondrial Complex I in Acquired Resistance to Trastuzumab

**DOI:** 10.3390/ijms25137397

**Published:** 2024-07-05

**Authors:** Ivana J. Tapia, Davide Perico, Virginia J. Wolos, Marcela S. Villaverde, Marianela Abrigo, Dario Di Silvestre, Pierluigi Mauri, Antonella De Palma, Gabriel L. Fiszman

**Affiliations:** 1 Universidad de Buenos Aires, Instituto de Oncología Ángel H. Roffo, Área de Investigación, 5481 San Martín Av., Ciudad Autónoma de Buenos Aires C1417DTB, Argentina; jwolos@institutoroffo.uba.ar (V.J.W.); marcelavillaverde@hotmail.com (M.S.V.); mabrigo@institutoroffo.uba.ar (M.A.); gfiszman@gmail.com (G.L.F.); 2 Institute of Biomedical Technologies-National Research Council ITB-CNR, Via Fratelli Cervi 93, 20054 Segrate, Italy; davide.perico@cnr.it (D.P.); dario.disilvestre@itb.cnr.it (D.D.S.); pierluigi.mauri@itb.cnr.it (P.M.); 3 Consejo Nacional de Investigaciones Científicas y Técnicas (CONICET), Ciudad Autónoma de Buenos Aires C1425FQB, Argentina; 4 Institute of Life Sciences, Sant’Anna School of Advanced Study, 56127 Pisa, Italy

**Keywords:** HER2+ breast cancer, Trastuzumab resistance, 3D cell culture, proteomics, systems biology, mitochondria

## Abstract

HER2-targeted therapies, such as Trastuzumab (Tz), have significantly improved the clinical outcomes for patients with HER2+ breast cancer (BC). However, treatment resistance remains a major obstacle. To elucidate functional and metabolic changes associated with acquired resistance, we characterized protein profiles of BC Tz-responder spheroids (RSs) and non-responder spheroids (nRSs) by a proteomic approach. Three-dimensional cultures were generated from the HER2+ human mammary adenocarcinoma cell line BT-474 and a derived resistant cell line. Before and after a 15-day Tz treatment, samples of each condition were collected and analyzed by liquid chromatography–mass spectrometry. The analysis of differentially expressed proteins exhibited the deregulation of energetic metabolism and mitochondrial pathways. A down-regulation of carbohydrate metabolism and up-regulation of mitochondria organization proteins, the tricarboxylic acid cycle, and oxidative phosphorylation, were observed in nRSs. Of note, Complex I-related proteins were increased in this condition and the inhibition by metformin highlighted that their activity is necessary for nRS survival. Furthermore, a correlation analysis showed that overexpression of Complex I proteins NDUFA10 and NDUFS2 was associated with high clinical risk and worse survival for HER2+ BC patients. In conclusion, the non-responder phenotype identified here provides a signature of proteins and related pathways that could lead to therapeutic biomarker investigation.

## 1. Introduction

Human epidermal growth factor receptor 2 (HER2) overexpression determines a subtype of mammary tumors that accounts for approximately 15% to 20% of primary breast cancers (BCs) [1]. This receptor belongs to the epidermal growth factor receptor (EGFR) family and contains an extracellular ligand-binding domain, a transmembrane domain and an intracellular tyrosine kinase domain. Normally, ligand binding to EGFRs results in their homodimerization or heterodimerization, leading to the activation of downstream signaling pathways. HER2 acts as an oncogene since its overexpression triggers ligand-independent receptor dimerization and abnormal signaling that promotes cell division and inhibits apoptosis [2].

Although HER2-positive (HER2+) tumors were initially associated with poor prognosis [3], knowledge about their etiology has enabled the development of targeted therapies that markedly improved survival outcomes. The first and the most widely used approach is based on Trastuzumab (Tz) [4], a humanized monoclonal antibody that binds to the extracellular domain of HER2 and hence suppresses intracellular signaling pathways, promotes cell cycle arrest, and triggers antibody-dependent cell-mediated cytotoxicity (ADCC) [5]. Driven by the therapeutic success achieved with Tz, other innovative drugs such as Pertuzumab [6], T-DM1 [7], Lapatinib [8], and Tz-deruxtecan [9] have been implemented in clinics in order to develop increasingly effective therapies. Currently, the standard of care for HER2+ BC patients involves the use of receptor-targeted drugs, as single agents or in a combination setting, in addition to chemotherapy and surgery [10].

Despite all these advances in targeting HER2 and increasing the survival of patients with this BC subtype, a substantial number of them develop therapeutic resistance and disease relapse [11]. In this regard, the large body of evidence that supports tentative mechanisms of resistance to Tz fall into two broad categories: extrinsic to the cancer cells, as immune-mediated mechanisms [12,13,14], and intrinsic to the tumor cells. The latter can be summarized as follows: (i) impaired binding of Tz to HER2, due to expression of receptor variants [15] or molecular masking [16]; (ii) interaction of HER2 with other dimerization partners, such as HER-family receptors [17] or insulin-like growth factor-1 receptors 1 and 2 (IGFR 1/2) [18]; (iii) constitutive activation of the PI3K/AKT/mTOR axis [19]; (iv) activation of alternative signaling pathways [20]; (v) increased ability to generate cancer stem cells [21]; (vi) vascular mimicry [22], hypoxia [23], and autophagy [24]; and (vii) metabolic adaptation [25,26,27,28]. Further exploration of these mechanisms is required to improve the clinical approach of this specific group of BC patients.

In this scenario, tumor resistance has been classically investigated using cancer cell lines cultured as monolayers. However, these results can only be partially translated into experimental outcomes in vivo [29] since these in vitro assays do not replicate either cell–extracellular matrix interactions or oxygen, nutrient, and pH gradients. These aspects of the tumor microenvironment play a crucial role in tumor progression, chemoresistance, and metastatic dissemination. Multicellular tumor spheroids are scaffold-free self-assembled aggregates of cancer cells which recapitulate some key features of solid malignancies. In fact, 3D cell cultures represent an in vitro self-organized and stimulus-sensitive model of an in vivo tissue [30]. Although this model only resembles the avascular region of in vivo tumor tissues [31], it is considered more realistic than monolayer cultures, exhibits repeated reproducibility, does not involve ethical concerns, and requires lower costs as compared to animal models. Overall, these features make them a suitable three-dimensional (3D) in vitro model for cancer research [21,24].

To further study these 3D cell culture models, mass spectrometry (MS)-based proteomics with the support of systems biology is becoming essential. Indeed, it is a valuable tool for the molecular characterization of specific pathophysiological conditions with diagnostic, prognostic, and therapeutic purposes [32,33]. Particularly, proteomics performed with nano-liquid chromatography coupled to high-resolution tandem mass spectrometry (nLC-hrMS/MS) [34,35] represents a powerful tool to explore the molecular mechanisms of pathologies and the cellular alterations induced by diseases, therapies, or environmental factors [36,37], and to identify potential biomarkers [38,39]. As regards the latter, it is crucial to stratify target patient populations, evaluate response to treatment, and customize therapeutic strategies, increasing their efficacy and reducing side effects.

In this study we characterized the proteomic profiles of tumor spheroids responder and non-responder to Tz, before and after treatment, using the HER2-overexpressing BT-474 human BC cell line in a 3D culture model. The main goal was to elucidate functional and metabolic changes associated with the resistant phenotype to identify possible therapeutic biomarkers in HER2+ BC.

## 2. Results

### 2.1. Three-Dimensional Architecture and Growth Kinetics of BT-474 Spheroids

**Figure 1:**
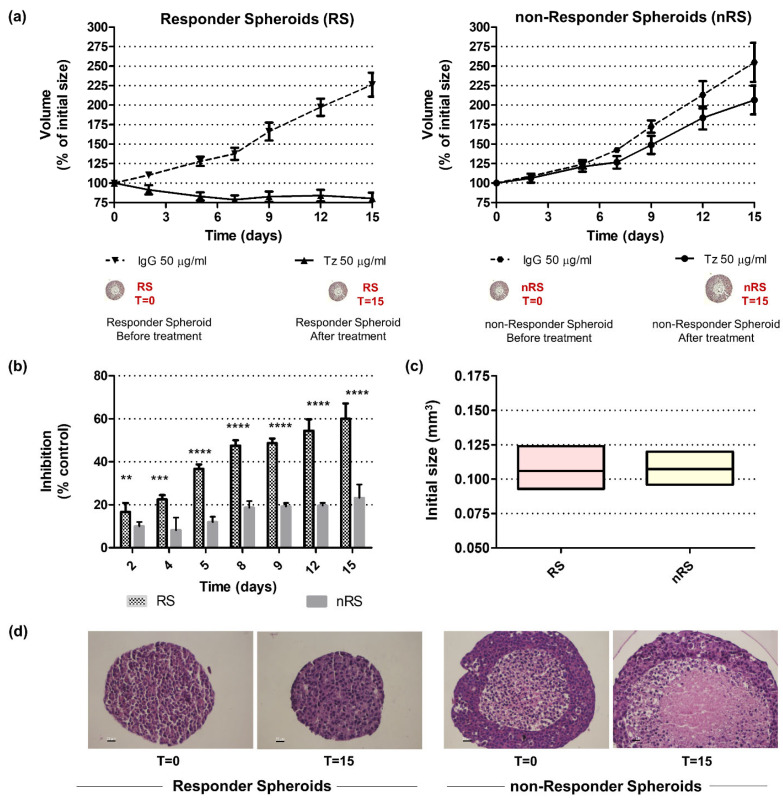
Three-dimensional architecture and growth kinetics of responder (RS) and non-responder (nRS) BT-474 tumor spheroids. RS and nRS were generated by the hanging-drop method using the HER2+ human mammary adenocarcinoma cell lines BT-474 and BT-474R, respectively. They were treated with 50 µg/mL Tz or 50 µg/mL IgG (control) for 15 days and their growth was followed by phase contrast microscope images. Volume was estimated from diameter determinations (Image Pro Plus 6), and it was normalized to the initial size. (**a**) Volume vs. time curves were created for both conditions (GraphPad 9.3.1). Representative graphs of three biological replicates are shown, mean ± SD. (**b**) Percentage of inhibition normalized to IgG at different times was calculated (two-way ANOVA—Sidak’s multiple comparisons test; *n* = 3). (**c**) Initial volume was estimated for each condition (paired *t*-test; *n* = 5). (**d**) Histology of the RSs and nRSs before and after treatment (hematoxylin–eosin staining; 20× magnification, scale = 25 µm). **, *** and ****, *p* < 0.01, *p* < 0.0005 and *p* < 0.0001, respectively.

In order to study acquired resistance to Trastuzumab (Tz), we performed 3D cell cultures from two HER2+ human breast adenocarcinoma cell lines: Tz-sensitive BT-474 and Tz-resistant BT-474 (BT-474R), which were obtained by chronic exposure to this antibody [17]. Thus, responder spheroids (RSs) and non-responder spheroids (nRSs) were generated using BT-474 and BT-474R cell lines, respectively. They were treated with 50 µg/mL Tz or immunoglobulin G (IgG), as control, for 15 days, and afterwards their volume was estimated. We considered the volume as a measure of spheroid growth since BT-474 cells are shaped as compact spheres. While RS slightly changed their volume after treatment, nRSs grew and doubled their initial volume (80.34% ± 7.40% and 206.52% ± 18.44%, respectively) (Figure 1a). Moreover, growth inhibition was significantly higher for RSs than for nRSs during the whole treatment (Figure 1b), indicating a differential pharmacological effect of Tz on spheroid growth.

Next, to corroborate the phenotype of remaining cells in RSs and nRSs after treatment, spheroids were disaggregated with trypsin-EDTA solution, single cells were seeded to grow as monolayers, and their sensitivity to Tz was assessed by dose–response curves. We found that cells derived from nRSs were still fully resistant to the antibody, whereas cells from RSs behaved as Tz-sensitive cells (% inhibition = 2.77% ± 2.19% and 59.28 ± 2.40%, respectively; *p* ˂ 0.0001, paired *t*-test, *n* = 4).

Interestingly, spheroids displayed differences in their 3D architecture, even if their initial volumes were similar (Figure 1c). RSs showed a homogeneous arrangement of cells which did not exhibit changes after treatment. In contrast, nRSs displayed a small central necrotic core: eosinophilic cells with poorly defined nuclei were surrounded by a rim of living cells with a high degree of compaction. An increase in the necrotic cell population compared to a thinner layer of living cells was suggested in nRSs after Tz treatment (Figure 1d).

Taken together, these results demonstrate that the 3D cell culture presented here constitutes a useful model to study Tz resistance since it mimics two clinical scenarios associated with a different response to the antibody. Moreover, RSs and nRSs exhibit dissimilar histological characteristics even before treatment, thus evidencing that they are not simple cellular aggregates, but partially recapitulate the complex and heterogeneous tumor organization, such as small avascular neoplasia or micrometastasis.

### 2.2. Proteomic Characterization of BT-474 Spheroids

In order to explore protein changes associated with responder and non-responder phenotypes, we carried out a proteomic analysis on BT-474 tumor spheroids using a shotgun label-free platform, based on the coupling of nano-liquid chromatography and high-resolution tandem mass spectrometry (nLC-hrMS/MS). Twenty-four proteomic profiles were acquired by the duplicate analysis of three biological replicates of four different conditions: RS and nRS before (RS T = 0 and nRS T = 0) and after 15 days (RS T = 15 and nRS T = 15) of treatment with Tz. An exemplary base peak chromatogram (BPC) of an analyzed sample (RS T = 0) is shown in Figure S1 of Supplementary Materials. A total of 3881 distinct protein groups with at least one unique peptide were identified and mapped to 3840 unique gene symbols, of which 2249 were present in at least two biological replicates in at least one group, ranging from 2.5 to 3900 kDa for the theoretical molecular weight and from 3.8 to 12.6 for the predicted isoelectric point (Table S1 of Supplementary Materials). For each experimental condition, a unique list was produced normalizing and averaging the peptide spectrum match values (aPSMs) attributed to the proteins, which represent the number of mass spectra assigned to each one and, indirectly, their abundance in the samples.

To assess the technical and biological repeatability of the samples analyzed, a correlation was tested with JMP 15.2 software. As shown in Figure S2 of Supplementary Materials, Pearson correlation coefficient (r) values indicated both high technical and biological repeatability. The distribution of the identified proteins (with a minimum frequency of 2 in at least one group) among the different conditions was visualized through a Venn diagram (Figure S3 of Supplementary Materials) created with InteractVenn [40]. The graph shows that most proteins were shared among the four groups and only a few were exclusively present in one specific condition, suggesting a quantitative rather than a qualitative difference in the proteomic profiles.

**Figure 2:**
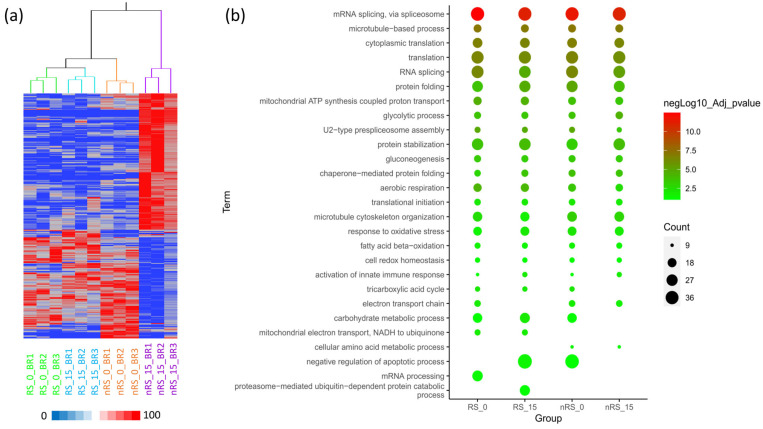
Hierarchical cluster and functional enrichment of the proteomic profiles for the analyzed conditions. In panel (**a**) the dendrogram was obtained by computing the peptide spectrum matches (PSMs) of statistically significant proteins selected by linear discriminant analysis (LDA); Euclidean distance metrics and Ward’s method were applied. The corresponding value of each protein is the average PSM (aPSM) of the two technical replicates of each biological replicate (BR1, BR2, and BR3). The color scale of the heatmap represents the range of the aPSM for each protein (from the minimum values in blue to the maximum values in red). The heatmap shows a good separation of the four different groups: RS T = 0 (green), RS T = 15 (light blue), nRS T = 0 (orange), and nRS T = 15 (purple). In panel (**b**), the functional enrichment analysis in biological processes for the four different conditions was performed with the DAVID database, and the resulting GO terms, filtered for adjusted *p*-value confidence and gene count, was plotted with ImageGP. The size of circles represents the number of genes associated with a GO term, while the color scale represents the negative log of the adjusted *p*-value as the confidence for the association.

The application of linear discriminant analysis (LDA) [41] with JMP 15.2 software on the total list of identified proteins allowed the extraction of 796 statistically significant proteins (F ratio > 3 and *p* < 0.05), able to stratify the different groups (Table S2 of Supplementary Materials). The hierarchical clustering performed on this list of proteins showed a good stratification of the samples (Figure 2a). In particular, two main clusters were identified, with the nRS T = 15 group presenting a completely different profile in protein expression compared to the other groups, which are more similar to each other and present a subgrouping for the responder condition (RS T = 0 and RS T = 15) separated from the non-responder one. This suggests that the first characteristic of the group’s separation is the response to Tz and not the treatment time. Moreover, the distinct molecular profile of nRS T = 15 is consistent with the different histological features that these spheroids exhibited, as described above (Figure 1d).

The list of statistically significant proteins was investigated by functional enrichment analysis to characterize the biological functions involved in the different groups. Specifically, Gene Ontology (GO) term enrichment on biological processes was performed using the DAVID database [42,43] and the results were plotted using the GO enrichment tool of the ImageGP platform [44], as shown in Figure 2b. The major enriched biological processes involved genetic information processing (translation and RNA splicing), aerobic metabolism (mainly mitochondrial and redox-related terms), and protein-related terms (protein stabilization and protein folding). Since the differences in gene count and confidence among different groups are very slight, changes in proteomic profiles may be attributable to a differential quantitative expression of a common group of proteins rather than the presence of proteins exclusive to a condition, as already suggested by the Venn diagram (Figure S3 of Supplementary Materials). Nevertheless, if we considered the proteins identified exclusively in RS and nRS groups, we were able to infer biological functions possibly related to the histological differences between the two conditions. In this way, through Voronoi graphs (Figure S4 of Supplementary Materials), based on KEGG gene classification, it was possible to observe a major involvement of carbon metabolism, transcription, and translation in RS groups, while pathways related to energy metabolism, signal transduction and cell growth were more abundant in nRSs.

### 2.3. Identification of Differentially Expressed Proteins and Network Analysis

To quantitatively examine the proteomic changes and to understand the possible mechanisms associated with the acquisition of resistance to Tz, a comparative analysis was performed to extract differentially expressed proteins (DEPs) among the examined conditions, starting from the list of statistically significant proteins by LDA (796 proteins). Using the home-made tool MAProMa and applying its two algorithms, DAve (Differential Average) and DCI (Differential Confidence Index), on the aPSM of each protein between two compared terms, it was possible to identify DEPs as up- and down-regulated proteins for the selected comparison.

Since the primary differences related to Tz resistance should be investigated before the treatment, and considering that nRS T = 15 showed a widely divergent molecular profile (as highlighted in Figure 2a), we focused on the comparisons between RS T = 0 and nRS T = 0 and between RS T = 0 and RS T = 15, allowing the extraction of 235 DEPs (Table S3 of Supplementary Materials). Specifically, 83 proteins were up-regulated and 55 were down-regulated in nRS T = 0 compared to RS T = 0, while 94 proteins were up-regulated and 56 were down-regulated in RS T = 15 compared to RS T = 0.

As DEPs may be related to biological functions associated with resistance to Tz, we further investigated their role. Thus, a protein–protein interaction (PPI) network based on both known and predicted interactions was built starting from the list of 235 DEPs using the STRING database [45]. The PPI network constituted of 233 nodes and 788 edges was then visualized and edited by Cytoscape 3.10.1 [46]. Specifically, in the PPI network, proteins are represented as nodes grouped in sub-networks based on their molecular function retrieved by the STRING enrichment Cytoscape plug-in. To show the differential expression of proteins in the different groups, two versions of the same network are depicted in Figure S5 of Supplementary Materials, representing the comparisons between RS T = 0 and nRS T = 0 groups (Figure S5a) and between RS T = 0 and RS T = 15 groups (Figure S5b).

**Figure 3:**
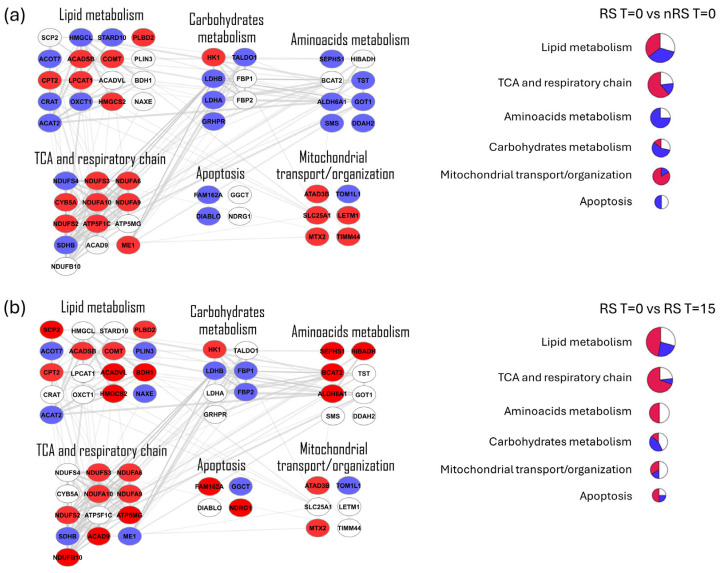
Network analysis. Protein–protein interaction (PPI) sub-networks of the differentially expressed proteins (DEPs) extracted from the comparisons between RS T = 0 and nRS T = 0 (**a**) and RS T = 0 and RS T = 15 (**b**) groups, related to energetic and mitochondrial-related pathways. In each network, red and blue nodes represent up-regulated and down-regulated proteins, respectively, while white nodes represent proteins that are not DEPs in that comparison. In panel (**a**), red nodes indicate proteins more expressed in nRS T = 0, while blue nodes indicate proteins more expressed in RS T = 0. In panel (**b**), red nodes indicate proteins more expressed in RS T = 15, while blue nodes indicate proteins more expressed in RS T = 0. For each sub-network, pie charts summarizing expression trends are depicted on the right. As for networks, red slices represent up-regulated proteins (more expressed in nRS_0 for panel (**a**) and more expressed in RS_15 for panel (**b**)), blue slices represent down-regulated proteins (more expressed in RS_0 in both panels), while white slices represent proteins that are not DEPs in the comparison. The size of the pie charts is proportional to the number of nodes belonging to a functional module in the sub-networks.

At first glance, it is possible to observe that several pathways have a similar trend in both comparisons, indicating a common deregulation. Particularly interesting is the up-regulation in nRS T = 0 of mitochondrial-related pathways, which may suggest a metabolic shift in the non-responder condition. Hence, for the two comparisons, the sub-networks of energetic and mitochondrial-related pathways were isolated and depicted in Figure 3, along with schematic representations of the expression trends through pie charts. The comparison between RS T = 0 and nRS T = 0 (Figure 3a) highlighted a higher expression of tricarboxylic acid cycle (TCA) and respiratory chain and mitochondrial transport/organization in nRS T = 0, while carbohydrate metabolism, amino acid metabolism, and apoptosis were more expressed in RS T = 0. The comparison between RS T = 0 and RS T = 15 (Figure 3b) highlighted a higher expression of lipid metabolism, TCA and respiratory chain, amino acid metabolism, and apoptosis in RS T = 15, while carbohydrate metabolism was more expressed in RS T = 0.

### 2.4. Functional Validations Suggest Mitochondrial Complex I Involvement

Down-regulation of carbohydrate metabolism and up-regulation of TCA and the respiratory chain in nRSs suggest changes related to cancer-specific energy production and carbon metabolism preference. Accordingly, we hypothesize that nRSs depend on aerobic energy metabolism sustained by an increase in mitochondrial activity, while the responder condition strongly relies on aerobic glycolysis.

**Figure 4:**
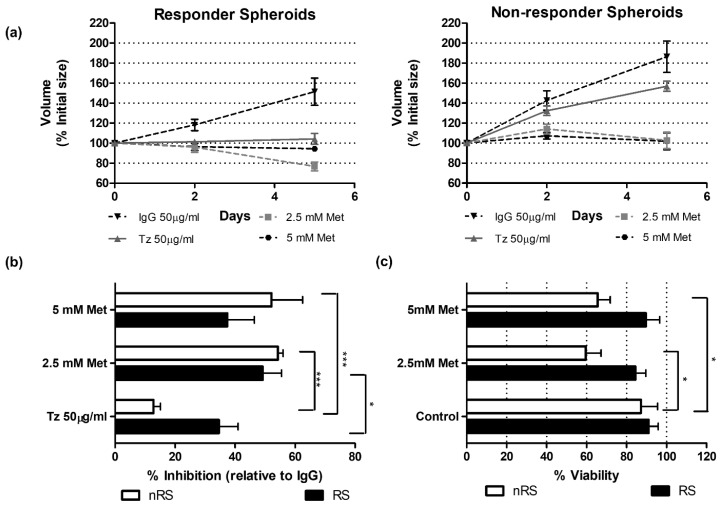
Effect of metformin, a Complex I inhibitor, on the growth kinetics and viability of responder (RS) and non-responder spheroids (nRS). (**a**) RSs and nRSs were treated for 5 days with 50 µg/mL Tz, 50 µg/mL IgG (control), 2.5 mM Met, or 5 mM Met, and volume vs. time curves were created for both conditions (GraphPad 9.3.1). Representative graphs of three biological replicates are shown, mean ± SD. (**b**) Percentage of inhibition normalized to IgG at different times was calculated (two-way ANOVA—Sidak’s multiple comparisons test; *n* = 4). (**c**) Spheroids were disaggregated and cell viability was estimated (trypan blue exclusion method) after a five-day treatment (two-way ANOVA—Sidak’s multiple comparisons test; *n* = 3). * and ***, *p* < 0.05 and *p* < 0.001, respectively. Tz: Trastuzumab, Met: metformin.

Since mitochondrial Complex I proteins are among the major mitochondrial-related DEPs, we analyzed the pharmacological inhibition of this complex to functionally validate the proteomic results. RSs and nRSs were treated with 2.5 mM or 5 mM metformin, and growth inhibition and cell viability were determined. Both conditions were sensitive to a five-day exposure to 2.5 mM metformin (Figure 4a), as RS and nRS growth was significantly inhibited by metformin treatment compared to Tz treatment (Figure 4b). This observation suggests that Complex I inhibition affects spheroid growth, although this is not exclusively associated with the non-responder condition.

Given that the abovementioned differences regarding the architecture of RSs and nRSs at baseline contribute to their volume (Figure 1d) in addition to growth inhibition, we evaluated the effect of metformin on spheroid viability. To do this, RSs and nRSs were disaggregated after a five-day treatment and cell viability was assessed by the trypan blue exclusion method. A significant reduction in nRS viability was observed after 2.5 mM and 5 mM metformin treatment, while RS viability was not affected (Figure 4c).

**Figure 5:**
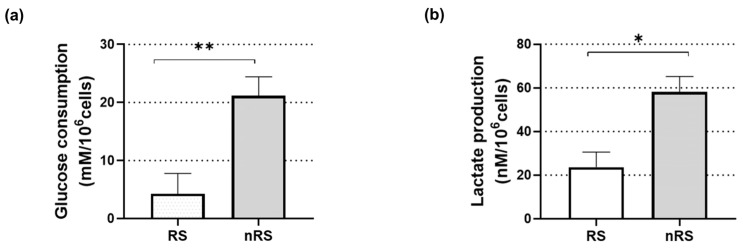
Lactate production and glucose consumption in responder and non-responder BT-474 tumor spheroids. Responder and non-responder spheroid culture supernatants were collected at baseline. (**a**) Glucose consumption and (**b**) lactate production were determined. Results were normalized to the number of total cells (two-way ANOVA—Sidak’s multiple comparisons test; *n* = 3). * and **, *p* < 0.05, *p* < 0.01, respectively.

In line with our hypothesis, we decided to further evaluate the counterpart of mitochondrial metabolism, lactate production, and glucose consumption as hallmarks of aerobic glycolysis. To achieve this goal, we collected the culture supernatant of BT-474 spheroids before Tz treatment (T = 0), determined lactate and glucose concentration, and finally, normalized the obtained values to the number of cells that comprised the spheroids. Lactate dehydrogenase catalyzes the interconversion of pyruvate and lactate with concomitant interconversion of NADH/NAD+, and is composed of two different subunits, LDHA and LDHB [47]. Since a down-regulation of both subunits was observed in the non-responder condition (Figure 3a), we expected a lower lactate production and glucose consumption compared to in the responder condition. Contrary to expectations, nRSs displayed enhanced lactate production and glucose uptake (Figure 5). This finding strongly suggests that, despite the increase in oxidative phosphorylation (OXPHOS)-related genes, nRSs depend on glycolysis for energy production.

**Figure 6:**
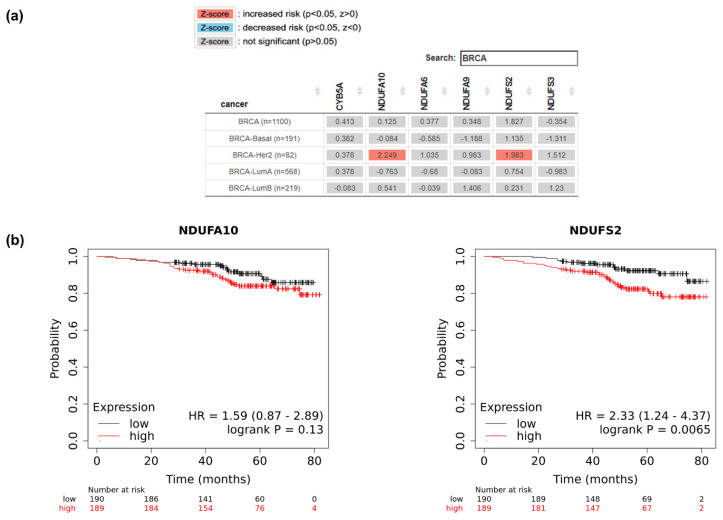
NDUFA10 and NDUFS2 expression correlates with poor survival in HER2+ breast cancer patients. The association between Complex I-related DEPs obtained from the network analysis and clinical outcome in breast cancer patients was assessed using (**a**) TIMER2.0 and (**b**) Kaplan–Meier plotter online tools.

In order to understand the clinical relevance of the Complex I increase in the resistant phenotype, we examined associations between DEPs related to this complex and patient clinical outcome. Tumor mRNA expression data collected from patients corresponding to different BC subgroups in The Cancer Genome Atlas dataset were first analyzed using the TIMER2.0 tool [48]. As shown in Figure 6a, this analysis revealed that two proteins that belong to Complex I (NDUFA10 and NDUFS2) are exclusively associated with a higher clinical risk (Z-score) in HER2-overexpressing tumors. Furthermore, using the Kaplan–Meier plotter tool [49], we found that patients with HER2+ tumors and high NDUFS2 mRNA levels had significantly poorer survival compared to the low-expression group (Figure 6b).

Taken together, these results demonstrate that metformin preferentially affects the viability of the nRSs but not of the RSs, functionally validating a mitochondrial Complex I activity increase in the non-responder condition. However, this rise does not correlate with a preponderance of mitochondrial activity in energetic terms, since aerobic glycolysis is increased in nRS. Interestingly, enhanced expression levels of Complex I proteins were associated with a higher risk and poorer survival in patients with HER2+ tumors. This leads us to speculate that this phenotype would have an impact on the clinical response, although the mechanism remains unknown.

## 3. Discussion

In this study, we carried out a proteomic analysis on HER2-overexpressing human breast cancer (BC) spheroids, as responders and non-responders to Trastuzumab (Tz), to investigate the proteome remodeling associated with acquired resistance to this drug in our 3D cell model. Using a shotgun label-free approach, several proteins were characterized to be differentially expressed in the context of Tz resistance, and correlated up- or down-regulated pathways were described.

The most relevant deregulated pathways regarded energetic metabolism and mitochondria. As highlighted in network analysis (Figure 3), the tricarboxylic acid cycle (TCA) and oxidative phosphorylation (OXPHOS) were significantly up-regulated in the non-responder condition. In addition, down-regulation of apoptosis and up-regulation of mitochondrial transport were observed. On the other hand, carbohydrate metabolism, including glycolysis, was more expressed in the responder condition.

A large body of evidence regarding changes affecting cancer cell metabolism has been described in the literature [50]. While energy production in normal cells derives mainly from OXPHOS, glycolysis is the main source of ATP supply in tumor cells, even in the presence of oxygen. This phenomenon is known as the Warburg effect [51] and, despite being less efficient than OXPHOS, it satisfies the high energy demand that implies increased proliferative activity. Previous reports described this phenotype for HER2-overexpressing tumors and possible mechanisms were suggested. Zhao and colleagues provided direct evidence supporting a causal relationship between HER2-overexpression and glycolysis in human BC cells [52], as it increases LDHA expression and its activity through the up-regulation of Heat Shock Transcription Factor 1 (HSF1); furthermore, Tz treatment inhibits lactate production and glucose uptake [53]. However, resistant cells are able to maintain glycolysis through the HSF1/LDHA axis even in the presence of this drug, and the combination of Tz with glycolysis inhibitors has been shown to overcome resistance [25].

The observed up-regulation of mitochondria-related proteins and the down-regulation of carbohydrate metabolism let us hypothesize that an OXPHOS-based metabolism is involved in Tz-acquired resistance instead of a Warburg-like phenotype. Glycolysis and mitochondrial OXPHOS are linked processes since the product of glycolysis, pyruvate, can either be converted to acetyl-CoA and enter the TCA or be transformed into lactic acid. Of note, we observed that the inhibition by metformin of mitochondrial Complex I, as part of OXPHOS, preferentially affected the viability of non-responder spheroids (Figure 4c). On the other hand, lactate production and glucose uptake in these spheroids were greater than in the responder condition (Figure 5), suggesting a key role of glycolysis for energy production rather than aerobic respiration. Although these results appear controversial, Gale and colleagues previously reported similar findings, based on RNA-seq analysis. They revealed that Tz-resistant cells up-regulated OXPHOS-related genes and were more sensitive to their blockade, as shown by ATP synthase inhibition, even though sensitive and resistant cells exhibited a similar respiration rate, ATP production, mitochondrial membrane potential, and oxygen consumption [26]. These authors speculated that Complex V is less efficient in the production of ATP in the resistant cells and that they functionally compensate for it by increasing Complex V gene expression. This finding is in good agreement with the HSP70-dependent translocation of HER2 to mitochondria, which has been shown to directly affect its functions in terms of complex activity and ATP production [27]. Furthermore, this translocation would cause a decrease in apoptosis, as we observed in the functional analysis (Figure 3a) and it is consistent with a resistant phenotype. Collectively, these data support the lack of correlation between the increase in OXPHOS-related proteins and an OXPHOS-based metabolism in the non-responder condition herein observed.

As described above, in addition to Complex I-related proteins, mitochondrial organization and transport proteins were also increased (Figure 3a), leading us to hypothesize that mitochondria may be involved in Tz resistance beyond its energetic role. In fact, this organelle has been considered crucial for other cellular functions such as apoptosis and production of reactive oxygen species [54,55]. Moreover, in the context of dual resistance to HER2, a recent proteomic study applied to Tz- and Pz-resistant cell lines revealed changes in mitochondrial activity [56].

On the other hand, the correlation analysis presented here indicates that high expression of NDUFS2 and NDUFA10, part of Complex I, was associated with high clinical risk and poorer survival for patients with HER2-overexpressing tumors (Figure 6). Accordingly, a study conducted by Jeon and colleagues has shown that an elevated expression of OXPHOS-encoding genes, in particular genes of core, accessory, and assembly subunits of Complex I, including NDUFS2 and NDUFA10, correlates with poor prognosis in lung adenocarcinoma patients [57]. Although the specific mechanism by which an up-regulation of this complex may confer resistance remains unknown, we can hypothesize possible explanations. First, Complex I has a fundamental role in NADH oxidation, which is considered essential to generate the membrane potential through the inner mitochondrial membrane that classically supports the synthesis of ATP [58]. Moreover, the maintenance of mitochondrial membrane potential also confers resistance to apoptosis by rendering it less sensitive to depolarization [59]. Indeed, it has been described that Complex I may act as a negative regulator of the mitochondrial permeability transition pore (mPTP), a fundamental player in the initiation of apoptosis and necrosis [60,61]. Consistently, our proteomic findings showed that the up-regulation of Complex I proteins is accompanied by a decrease in apoptosis and an increase in cell division (Figure S3), typical features of a resistant phenotype. In this sense, Complex I activity appears to be crucial for supporting cell proliferation, since it is related to aspartate production [58]. This amino acid is required for the synthesis of proteins, as well as purines and pyrimidines [62], and it is normally synthesized in the mitochondrial matrix through the sequential actions from malate to oxaloacetate by MDH2 and GOT2 enzymes and then transported to the cytosol. NAD+/NADH balance is fundamental for aspartate synthesis as MDH2 is an oxidoreductase. In contrast, it has been shown in an OXPHOS dysfunction system that aspartate formation is completely dependent on the activity of cytosolic GOT1 since it is mainly obtained from glutamine metabolism and the loss of this enzyme is lethal to cells [63]. Besides the increase in Complex I-related proteins, we observed a down-regulation of GOT1 in the Tz non-responder condition. This is consistent with the data reported above and it could explain why disrupting NAD balance by inhibiting Complex I affects spheroid viability (Figure 4c).

Finally, it has become apparent that metformin, as a Complex I inhibitor, could be a promising drug to overcome Tz resistance. Although in vitro and in vivo results were mostly encouraging [64,65], the common dose used was 10 to 1000 times higher than the maximum plasma levels observed in diabetic patients [66]. Therefore, the clinical relevance of metformin remains a topic of debate. A phase two clinical trial revealed that the addition of a conventional anti-diabetic dose of metformin in a neoadjuvant setting in women with early HER2+ BC was well tolerated and safe [67]. Further studies are needed to confirm whether metformin could provide a therapeutic opportunity for HER2+ BC patients.

## 4. Materials and Methods

### 4.1. BT-474 Tumor Spheroid Preparation and Treatment

#### 4.1.1. Drugs and Treatments

Tumor spheroids were treated with Trastuzumab (Herceptin, Genentech/Roche, San Diego, CA, USA) or an unrelated human IgG (UNC Hemoderivados, Córdoba, Argentina) as a control, at 50 μg/mL for 15 days. Metformin (Islotin, Craveri, Ciudad Autónoma de Buenos Aires, Argentina) was employed at two concentrations, 2.5 and 5 mM, for 5 days. The adequate concentration of each drug was added in each media replacement, performed every two days, and constantly maintained during the experiment.

#### 4.1.2. Cell Cultures and Generation of Tumor Spheroids

The human mammary adenocarcinoma BT-474 cell line was obtained from the American Tissue Culture Collection (ATCC, Manassas, VA, USA) and was grown in RPMI 1640 (Gibco, Thermo-Fisher Scientific, Waltham, MA, USA) supplemented with 10% fetal bovine serum (Internegocios S.A., Buenos Aires, Argentina) and 50 µg/mL gentamicin. Serial passages were carried out by treatment with 0.25% trypsin and 0.075% EDTA (Sigma-Aldrich Inc., St. Louis, MO, USA). Tz-resistant BT-474 cells (BT-474R) were obtained by continuous treatment of monolayers with Tz (10 μg/mL) for up to 6 months, as we reported previously [21]. Tz resistance was periodically tested using the colorimetric MTS assay (Cell Titer 96 non-radioactive cell proliferation assay kit, Promega Inc., Madison, WI, USA) to evaluate cell viability in the presence of an increasing concentration of Tz (0.001–50 µg/mL). Inhibition of cell proliferation was significantly lower for BT-474R than for BT-474 after 5 days of treatment with 10 µg/mL Tz (12.17% ± 8.89% and 56.33% ± 2.31%, respectively, *p* = 0.01, paired *t*-test; *n* = 3), confirming the resistant phenotype.

To generate spheroids, we adapted the hanging drop method [68]. Briefly, 1 × 10^4^ cells were seeded on the cover of 96-well plates in 20 μL drops. Covers were then inverted and incubated for 72 h until spheroids were fully formed, after which they were transferred into individual wells coated with 1.5% agarose and 200 μL of complete medium. Spheroids were fed every other day by carefully aspirating 100 μL of medium and replacing it with the same volume of fresh complete medium. To evaluate Tz chronic treatment in 3D, experiments were performed when spheroids reached a diameter ≥ 550 μm, corresponding approximately to day 7.

#### 4.1.3. Tumor Spheroid Growth

Spheroids were treated as described before and their growth was followed by contrast microscope images. At least six individual spheroids were performed for each treatment. Volume was estimated from diameter determinations (Image Pro Plus 6) using the sphere formula and normalized to the initial size. Afterwards, volume versus time curves were generated (GraphPad 9.3.1). The percentage of inhibition normalized to *IgG* was calculated by applying the following formula:

$$\%\;Inhibition\;=\;100\;-\;\frac{Vol\;Tz\;\left(Day\;n\right)}{Vol\;IgG\;\left(Day\;n\right)}\times \;100$$

where:

*n* is the specific day after treatment.

*Vol Tz* is the average volume of spheroids treated with *Tz* at day *n*.

*Vol IgG* is the average volume of spheroids treated with *IgG* at day *n*.

#### 4.1.4. Tumor Spheroid Histology

Spheroids were collected at the end of each experiment. Subsequently, they were fixed in buffered formalin for 30 min, dehydrated in alcohol gradient, embedded in paraffin, and sectioned (5 μm thickness). These sections were deparaffinized in xylene, rehydrated in an alcohol gradient, and finally stained with hematoxylin–eosin (H&E) for histology analysis.

#### 4.1.5. Tumor Spheroid Viability

Spheroid viability after metformin treatment was analyzed by the trypan blue exclusion method (Thermo-Fisher Scientific, Waltham, MA, USA). Briefly, spheroids were disaggregated with 0.25% trypsin and 0.075% EDTA for 5 min at 37 °C and, afterward, total cell counts and cell integrity were determined using a Neubauer hemocytometer (Sigma-Aldrich Inc., St. Louis, MO, USA).

#### 4.1.6. Glucose Consumption and Lactate Production Determinations

Spheroid culture supernatants were collected at baseline and 5 µL of this was transferred to a new 96-well plate. Then, glucose and lactate concentrations were determined using commercial kits (Weiner Lab., Rosario, Argentina and Cobas/Roche, San Diego, CA, USA, respectively). Glucose consumption was estimated from the difference between moles of glucose in the cell-free culture medium and moles of glucose in the supernatant. Results were normalized to the total number of cells.

### 4.2. LC-MS/MS Analysis of Responder and Non-Responder BT-474 Tumor Spheroids

#### 4.2.1. In-Solution Digestion

Proteomic analysis was performed on three biological replicates of RS nRS BT-474 spheroids before (T0) and after 15 days (T15) of treatment with Tz (*n* = 24 samples). Spheroid samples were suspended in 0.1 M NH_4_HCO_3_ pH 7.9 and treated with RapiGest^TM^ SF reagent (Waters Co, Milford, MA, USA) at the final concentration of 0.25% (*w*/*v*). The resulting suspensions were incubated while stirring at 100 °C for 20 min. The digestion was carried out on each sample by adding Sequencing Grade Modified Trypsin (Promega Inc., Madison, WI, USA) at an enzyme/substrate ratio of 1:50 (*w*/*v*) overnight at 37 °C in 0.1 M NH_4_HCO_3_ pH 7.9 buffer with 10% CH_3_CN. An additional aliquot of trypsin (1:100 *w*/*v*) was added in the morning and the digestion continued for 4 h. Moreover, the addition of 0.5% Trifluoroacetic acid (TFA) (Sigma-Aldrich Inc., St. Louis, MO, USA) stopped the enzymatic reaction and a subsequent incubation at 37 °C for 45 min completed the RapiGest acid hydrolysis [69]. The water-immiscible degradation products were removed by centrifugation at 13,000 rpm for 10 min. Finally, the tryptic digest mixtures were desalted using Pierce^TM^ C-18 spin columns (Life Technologies, Segrate, Italy), according to the manufacturer’s protocol and were resuspended in 0.1% formic acid (Sigma-Aldrich Inc., St. Louis, MO, USA) in water (LC-MS Ultra CHROMASOLV^TM^, Honeywell Riedel-de Haen^TM^, Muskegon, MO, USA) at a concentration of 0.1 µg/µL.

#### 4.2.2. Liquid Chromatography

Peptide mixtures were analyzed using an Eksigent nanoLC-Ultra^®^ 2D System (Eksigent, part of AB SCIEX Dublin, CA, USA) combined with a cHiPLC-nanoflex system (Eksigent) in trap-elute mode. Briefly, for each condition, two technical replicates were performed, injecting 0.8 µg of proteins on the cHiPLC trap (200 µm × 500 µm ChromXP C18-CL, 3 µm, 120 Å, Eksigent, part of AB SCIEX, Dublin, CA, USA) and running the loading pump in isocratic mode with 0.1% formic acid in water for 10 min at a flow of 3 µL/min. The automatic switching of the cHiPLC ten-port valve then eluted the trapped mixture on a nano cHiPLC column (75 µm × 15 cm ChromXP C18-CL, 3 µm, 120 Å, Eksigent, part of AB SCIEX, Dublin, CA, USA) through a 135 min gradient of eluent B (eluent A, 0.1% formic acid in water; eluent B, 0.1% formic acid in acetonitrile) at a flow rate of 300 nL/min. In detail, the gradient was as follows: from 5–10% B in 3 min, 15–30% B in 110 min, 40–95% B in 12 min, and holding at 95% B for 10 min.

#### 4.2.3. Mass Spectrometry

The eluted peptides were directly analyzed on an Orbitrap Exploris 120 mass spectrometer (Thermo Fisher Scientific, San Josè, CA, USA) equipped with an EASY-Spray ion source (Thermo Fisher Scientific, San Josè, CA, USA). Easy spray was achieved using an EASY-Spray Emitter (Thermo Fisher Scientific, San Josè, CA, USA) (nanoflow 7 µm ID Transfer Line 20 µm × 50 cm) held to 1.6 kV, while the ion transfer capillary was held at 220 °C. Data-dependent acquisition (DDA) was performed, acquiring precursor ions in the *m*/*z* range of 375–1250 with a resolution (at *m*/*z* 200) of 60,000 FWHM (full width at half maximum). Precursor fragmentation was carried out at a resolution (at *m*/*z* 200) of 15,000 FWHM, using a higher-energy collisional dissociation (HCD) method with normalized collision energy (NCE) of 30 eV and a dynamic exclusion of 20 s. MS and MS/MS data were acquired in profile and centroid modes, respectively, using positive polarity and isotope exclusion set to on. The isolation width was set to 2 *m*/*z* and first mass was set to 120 *m*/*z*. Mass spectrometer scan functions and high-performance liquid chromatography solvent gradients were controlled by the Xcalibur data system version 4.4 (Thermo Fisher Scientific, CA, USA) and Eksigent Control Software version 4.3 (Eksigent, part of AB SCIEX, Dublin, CA, USA), respectively.

#### 4.2.4. Proteomic Data Processing and Data Mining

All generated data were searched using the Sequest HT search engine contained in the Thermo Scientific Proteome Discoverer software, version 2.1. The experimental MS/MS spectra were correlated to tryptic peptide sequences by comparison with the theoretical mass spectra obtained by in silico digestion of the UniProt Homo sapiens proteome database (75,550 entries) (www.uniprot.org, accessed on 23 March 2021). The following criteria were used for the identification of peptide sequences and related proteins: trypsin as enzyme, three missed cleavages per peptide, methionine oxidation as variable modification, mass tolerances of ±10 ppm for precursor ions and ±0.05 Da for fragment ions. Percolator node was used with a target-decoy strategy to give a final false discovery rate (FDR) at the peptide spectrum match (PSM) level of 0.01 (strict) based on q-values, considering a maximum deltaCN of 0.05 [70]. Only peptides with a minimum peptide length of six amino acids and rank 1 were considered. Protein grouping and the strict parsimony principle were applied. Additional filtering was applied to retain only proteins with high confidence, using peptide validator and peptide and protein filter nodes. The proteomic datasets of this study can be found in the MassIVE database at the link ftp://massive.ucsd.edu/v06/MSV000093670/ (accessed on 14 December 2023).

The 24 protein lists obtained from the SEQUEST algorithm were aligned, normalized and label-free compared. The protein lists were subjected to linear discriminant analysis (LDA) [41] with JMP 15.2 software (SAS Institute Inc., Cary, NC, USA) and proteins with the largest F ratio (≥3) and smallest *p*-value (<0.05) were retained and processed by hierarchical clustering, applying Ward’s method and the Euclidean distance metric using JMP 15.2 software. Specifically, the F ratio represented the model mean square divided by the error mean square, whereas the *p*-value indicated the probability of obtaining an F value greater than that calculated if there was no difference between the population group means.

Statistically discriminant proteins obtained from LDA were processed by an in-house algorithm, namely, the Multidimensional Algorithm Protein Map (MAProMa), using the average peptide spectrum matches (aPSMs) [71,72] that correspond to the average of all the spectra identified for a protein and, consequently, to its relative abundance, in each analyzed condition. In detail, to select differentially expressed proteins, the different groups were pairwise compared by applying a threshold of 0.35 (corresponding to a fold change of 1.5) and 4.5 on the two MAProMa indexes, DAve (Differential Average) and DCI (Differential Confidence Index), respectively. DAve, which evaluates changes in protein expression, was defined as (X − Y)/(X + Y)/0.5, while DCI, which evaluates the confidence of differential expression, was defined as (X + Y) × (X − Y)/2. The X and Y terms represent the PSM of a given protein in two compared samples.

Venn diagrams were created using the InteractiVenn website (InteractiVenn—Interactive Venn Diagrams) [40] on the list of proteins identified in at least two biological replicates in at least one group.

Gene Ontology (GO) enrichment analyses were performed using the DAVID database (https://david.ncifcrf.gov, accessed on 1 October 2023) [42,43]. The Select Identifier was set to “UNIPROT ACCESSION” and the species was set to “Homo sapiens.” Then, a biological process (BP) analysis was performed. The data were exported and sorted according to the Bonferroni adjusted *p*-value. For each GO enrichment, the items that exceeded the thresholds of gene count > 5 and Bonferroni *p*-value < 0.001 were selected to draw the advanced bubble diagram using the ImageGP platform [44] (https://www.bic.ac.cn/ImageGP/, accessed on 14 December 2023), using default settings, for the four different conditions.

Voronoi graphs from proteome data were built using the Proteomaps tool (available at www.proteomaps.net, accessed on 28 June 2024) [73], which is based on the KEGG pathway gene classification. Each biological function is shown by a polygon whose size is a function of the abundance of the proteins associated with that category, and functionally related proteins are arranged in common regions.

#### 4.2.5. Network Analysis

Starting from the list of differentially expressed proteins (DEPs) obtained by LDA and MAProMa results, a protein–protein interaction (PPI) network (233 nodes and 788 edges) was built by the STRING database [45,74]; only experimentally and database-defined PPIs with a score > 0.4 were considered. The resulting sub-network was visualized and analyzed by Cytoscape v. 3.10.1 and its plugins [46]. Proteins were grouped in functional modules with the support of STRING enrichment, using default settings and combining different sources such as Gene Ontology, KEGG pathways, and Reactome pathways.

### 4.3. Analysis of Clinical Data

The prognostic significance of the mRNA expression of mitochondrial Complex I genes in breast cancer was assessed using TIMER 2.0 (http://timer.cistrome.org) [48] and Kaplan–Meier Plotter (https://kmplot.com) [49] web tools. Briefly, in TIMER2.0, the Gene_Outcome module was used to evaluate the outcome significance of gene expression using the normalized coefficient of the genes in the Cox proportional hazard model. As regards Kaplan–Meier Plotter, the RNA-seq platform for breast cancer was used, restricted to the HER2+ subtype. Patients were divided into low- or high-expression groups according to the median expression of the genes, and Kaplan–Meier overall survival plots were obtained.

### 4.4. Statistical Analysis

Data are expressed as mean ± SD. All experiments were performed at least in triplicates. Statistical analyses were performed by the corresponding Student’s *t*-test or ANOVA test, and differences between groups were compared by Sidak’s post-test analysis, utilizing the GraphPad Prism 9.3.1 software. *p* values < 0.05 were considered significant.

## 5. Conclusions

The application of proteomics to a 3D mammary tumor model of responders and non-responders to Tz allowed us to explore the specific changes associated with acquired resistance to this antibody. Specifically, using this model we were able to focus on intrinsic tumor cell changes due to the absence of the vascular and immunological microenvironment. Spheroids were analyzed in their entirety and a comprehensive examination of their substructural complexity was beyond the aim of this work. Future studies may be focused on the characterization of different cell subpopulations, such as the rim and the necrotic core, possibly taking advantage of innovative single-cell techniques.

This study revealed a signature of proteins and related pathways involved in Tz resistance, and represents a good starting point to elucidate new specific mechanisms and, in particular, the role played by mitochondria. Likewise, this signature deserves in-depth studies for possible biomarkers or therapeutic targets, such as NDUFA10 and NDUFS2. The identification of these biomarkers will be crucial to stratify target patient populations, and a more complete understanding may be achieved by further evaluation in a cohort of patients with HER2+ BC, including the potential effect of metformin on Tz resistance. This could provide personalized therapeutic strategies, increasing the efficacy of drug treatment and reducing its side effects.

## Data Availability

The proteomic datasets (in form of raw data) analyzed for this study can be found in the MassIVE database at the link ftp://massive.ucsd.edu/v06/MSV000093670 (accessed on 14 December 2023).

## Supplementary Materials

The following supporting information can be downloaded at: https://www.mdpi.com/article/10.3390/ijms25137397/s1.
