# Supplementary figures and images for: Proteomic Characterization of a 3D HER2+ Breast Cancer Model Reveals the Role of Mitochondrial Complex I in Acquired Resistance to Trastuzumab
Source: Int J Mol Sci. 2024 Jul 5;25(13):7397. doi: 10.3390/ijms25137397 (PMC11242363; doi:10.3390/ijms25137397)

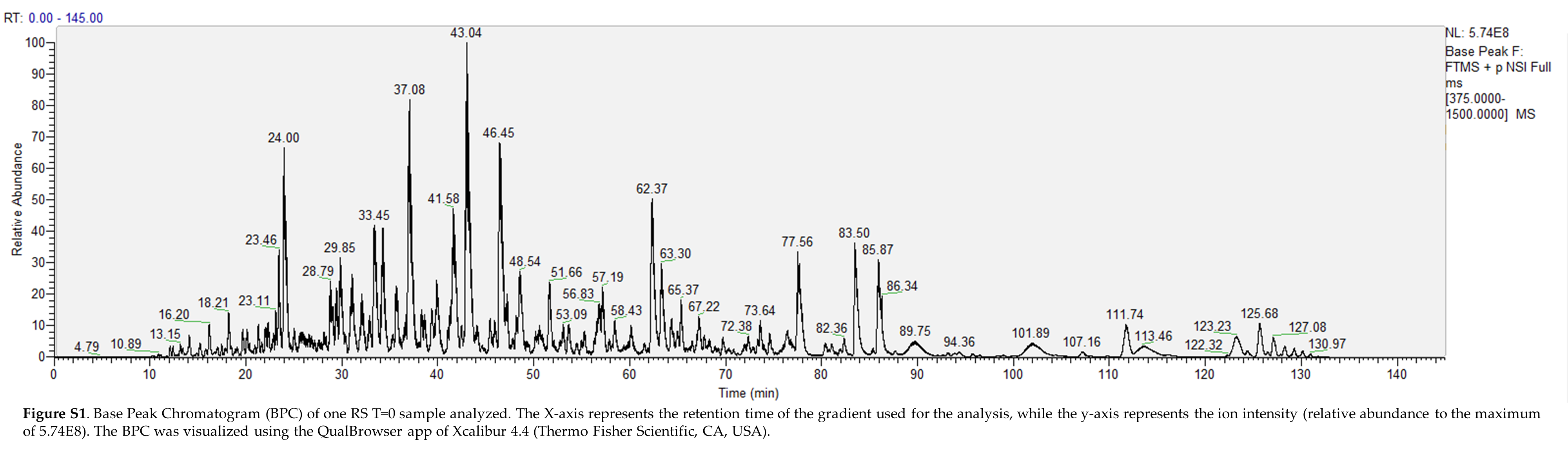

Supplement: Supplementary file 1 [file ijms-25-07397-s001.zip › Figure_S1.tif]

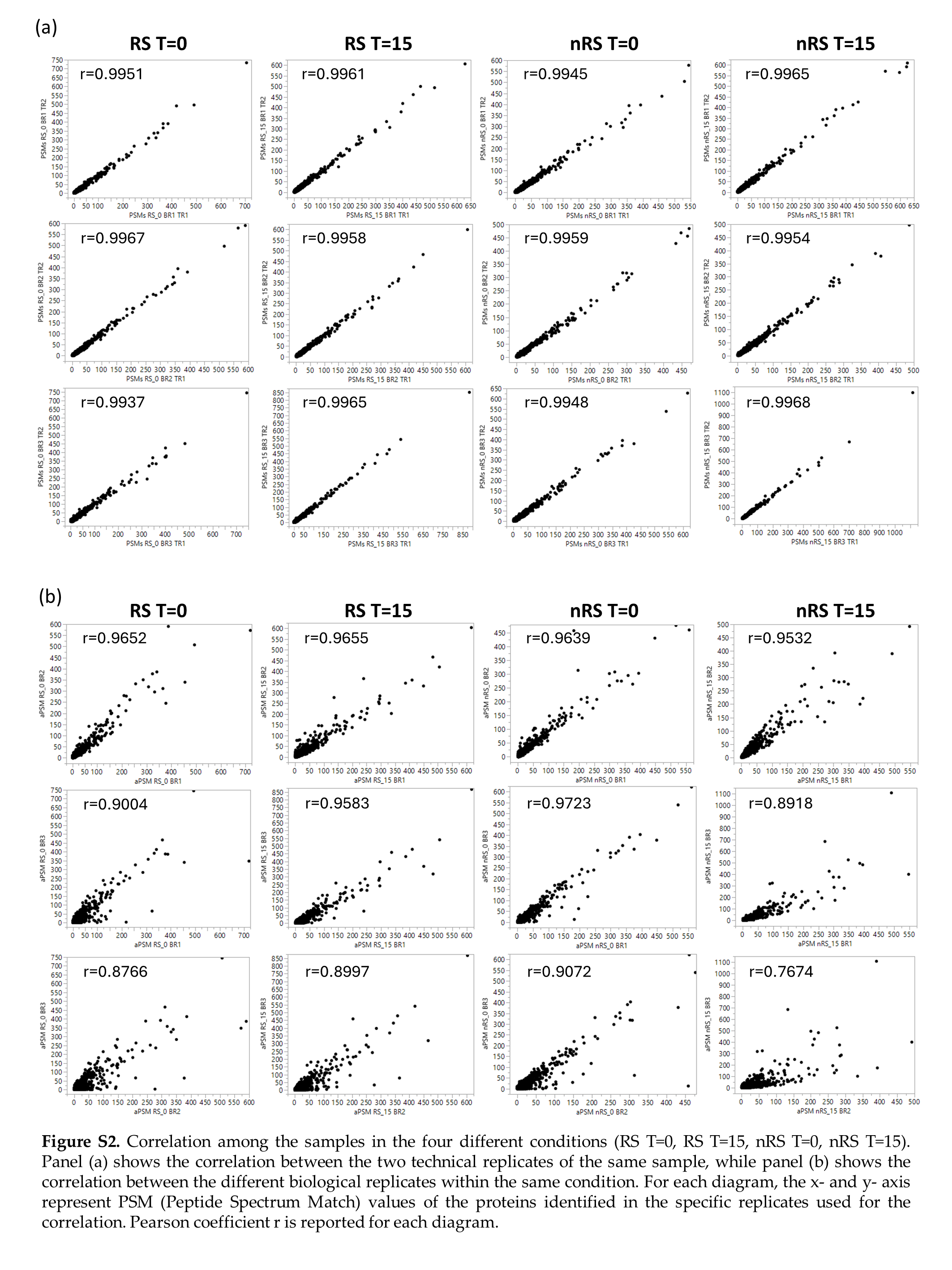

Supplement: Supplementary file 1 [file ijms-25-07397-s001.zip › Figure_S2.tif]

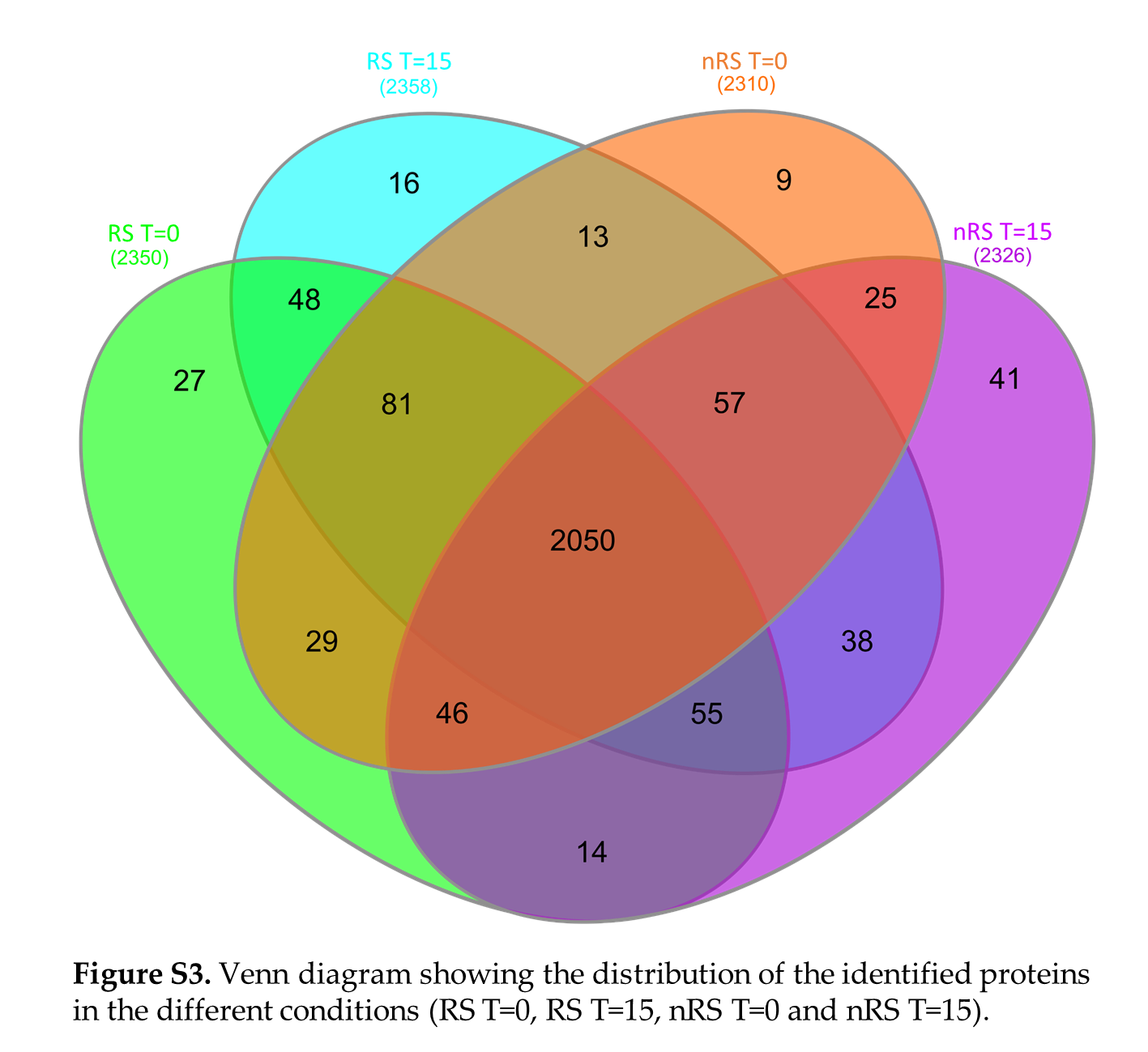

Supplement: Supplementary file 1 [file ijms-25-07397-s001.zip › Figure_S3.tif]

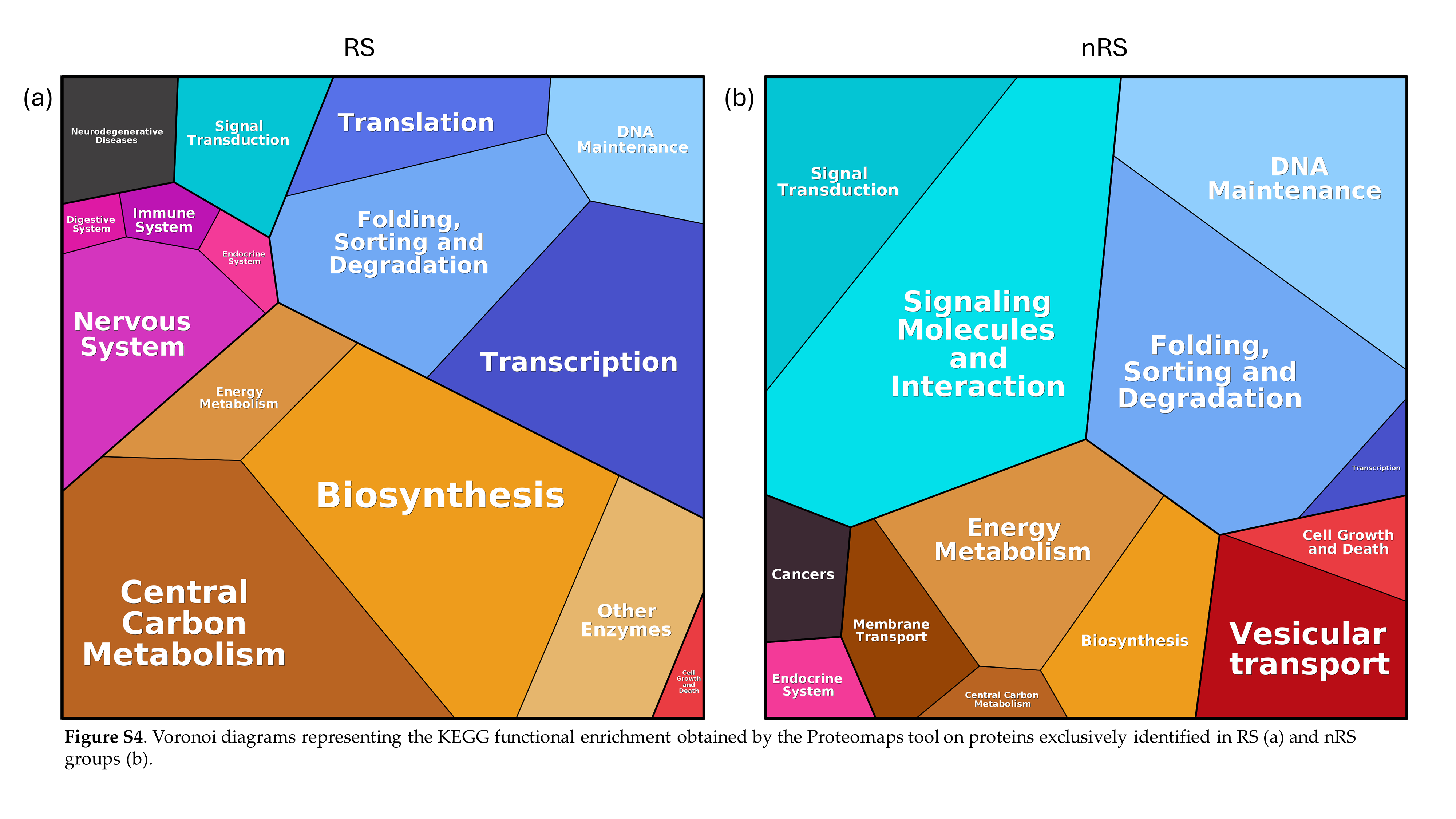

Supplement: Supplementary file 1 [file ijms-25-07397-s001.zip › Figure_S4.tif]

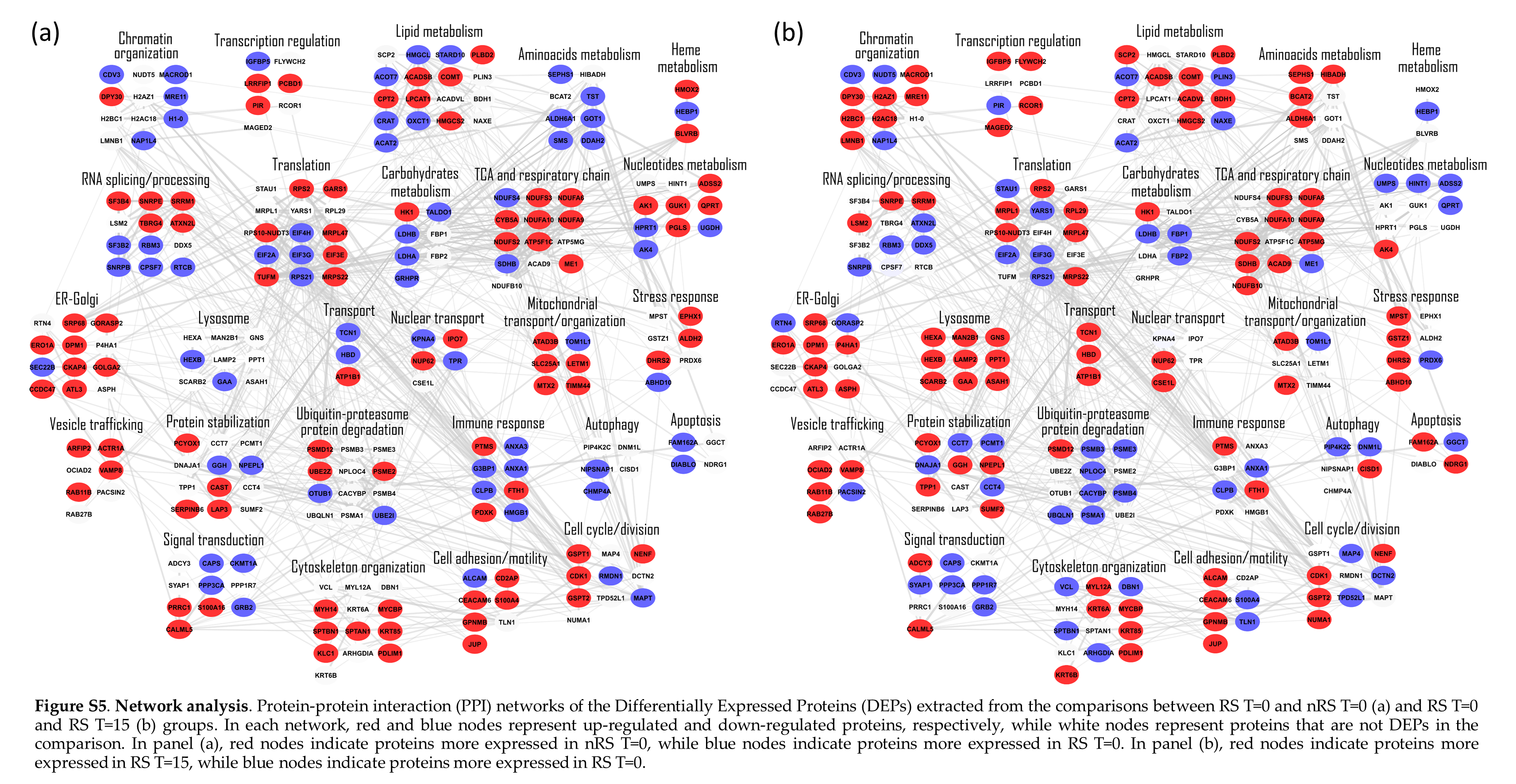

Supplement: Supplementary file 1 [file ijms-25-07397-s001.zip › Figure_S5.tif]
